# Biological Membrane-Penetrating Peptides: Computational Prediction and Applications

**DOI:** 10.3389/fcimb.2022.838259

**Published:** 2022-03-25

**Authors:** Ewerton Cristhian Lima de Oliveira, Kauê Santana da Costa, Paulo Sérgio Taube, Anderson H. Lima, Claudomiro de Souza de Sales Junior

**Affiliations:** ^1^ Institute of Technology, Federal University of Pará, Belém, Brazil; ^2^ Laboratory of Computational Simulation, Institute of Biodiversity, Federal University of Western Pará, Santarém, Brazil; ^3^ Laboratório de Planejamento e Desenvolvimento de Fármacos, Instituto de Ciências Exatas e Naturais, Universidade Federal do Pará, Belém, Brazil

**Keywords:** pharmacokinetics, machine learning, cell membrane, peptides, blood-brain barrier, structure activity, cell-penetrating peptides, drug system carriers

## Abstract

Peptides comprise a versatile class of biomolecules that present a unique chemical space with diverse physicochemical and structural properties. Some classes of peptides are able to naturally cross the biological membranes, such as cell membrane and blood-brain barrier (BBB). Cell-penetrating peptides (CPPs) and blood-brain barrier-penetrating peptides (B3PPs) have been explored by the biotechnological and pharmaceutical industries to develop new therapeutic molecules and carrier systems. The computational prediction of peptides’ penetration into biological membranes has been emerged as an interesting strategy due to their high throughput and low-cost screening of large chemical libraries. Structure- and sequence-based information of peptides, as well as atomistic biophysical models, have been explored in computer-assisted discovery strategies to classify and identify new structures with pharmacokinetic properties related to the translocation through biomembranes. Computational strategies to predict the permeability into biomembranes include cheminformatic filters, molecular dynamics simulations, artificial intelligence algorithms, and statistical models, and the choice of the most adequate method depends on the purposes of the computational investigation. Here, we exhibit and discuss some principles and applications of these computational methods widely used to predict the permeability of peptides into biomembranes, exhibiting some of their pharmaceutical and biotechnological applications.

## Getting Across the Biological Barriers: An Overview on the Scientific Significance and Current Knowledge

Penetration into biological membranes is a desired characteristic for bioactive molecules to reach their target site related to the molecular mode of action (Doak et al., 2014; Daina and Zoete, 2016). Molecules that naturally cross these biomembranes have been investigated aiming at different biotechnological and pharmaceutical applications (Rossi Sebastiano et al., 2018; Derakhshankhah and Jafari, 2018). The selective control performed by these biomembranes has protected the living organisms against undesired and harmful effects of exogenous molecules and the invasion of pathogens. However, these membranes have been the main challenge to developing new potent therapeutic compounds, and several strategies have been developed to overcome this obstacle (Bennion et al., 2017; Roy Chowdhury et al., 2018; Ahlawat et al., 2020). Two biomembranes have been the focus of the pharmaceutical and biotechnological industries: cell membrane and the blood-brain barrier (BBB) (Yang and Hinner, 2015; Oller-Salvia et al., 2016; Zhou et al., 2021).

**Graphical Abstract:**
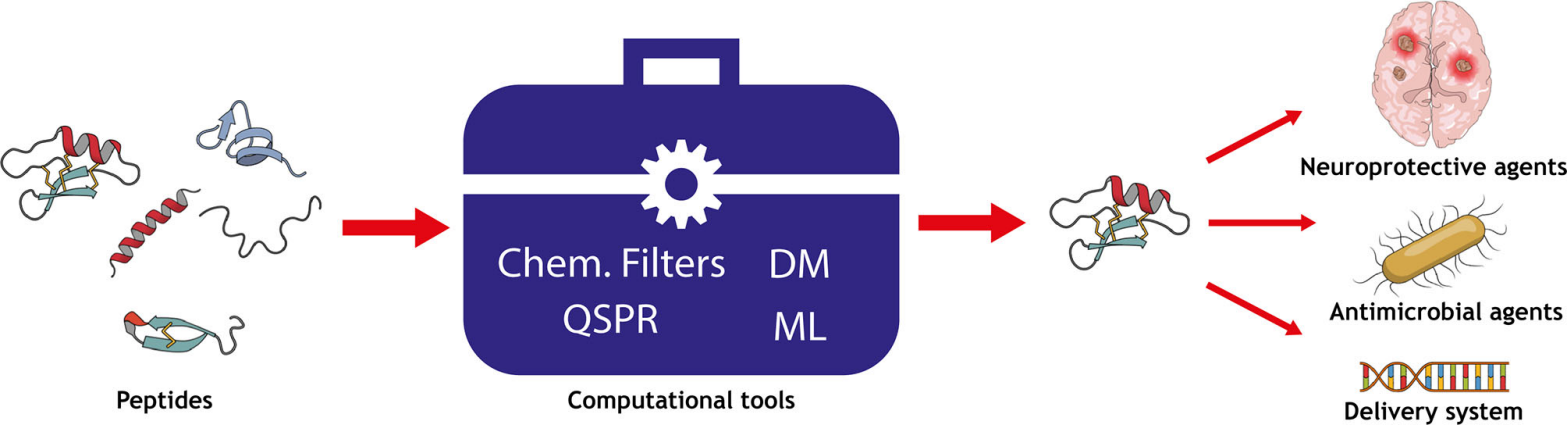
Overview of the computational techniques applied to predict the permeability of peptides into biomembranes.

**Figure 1:**
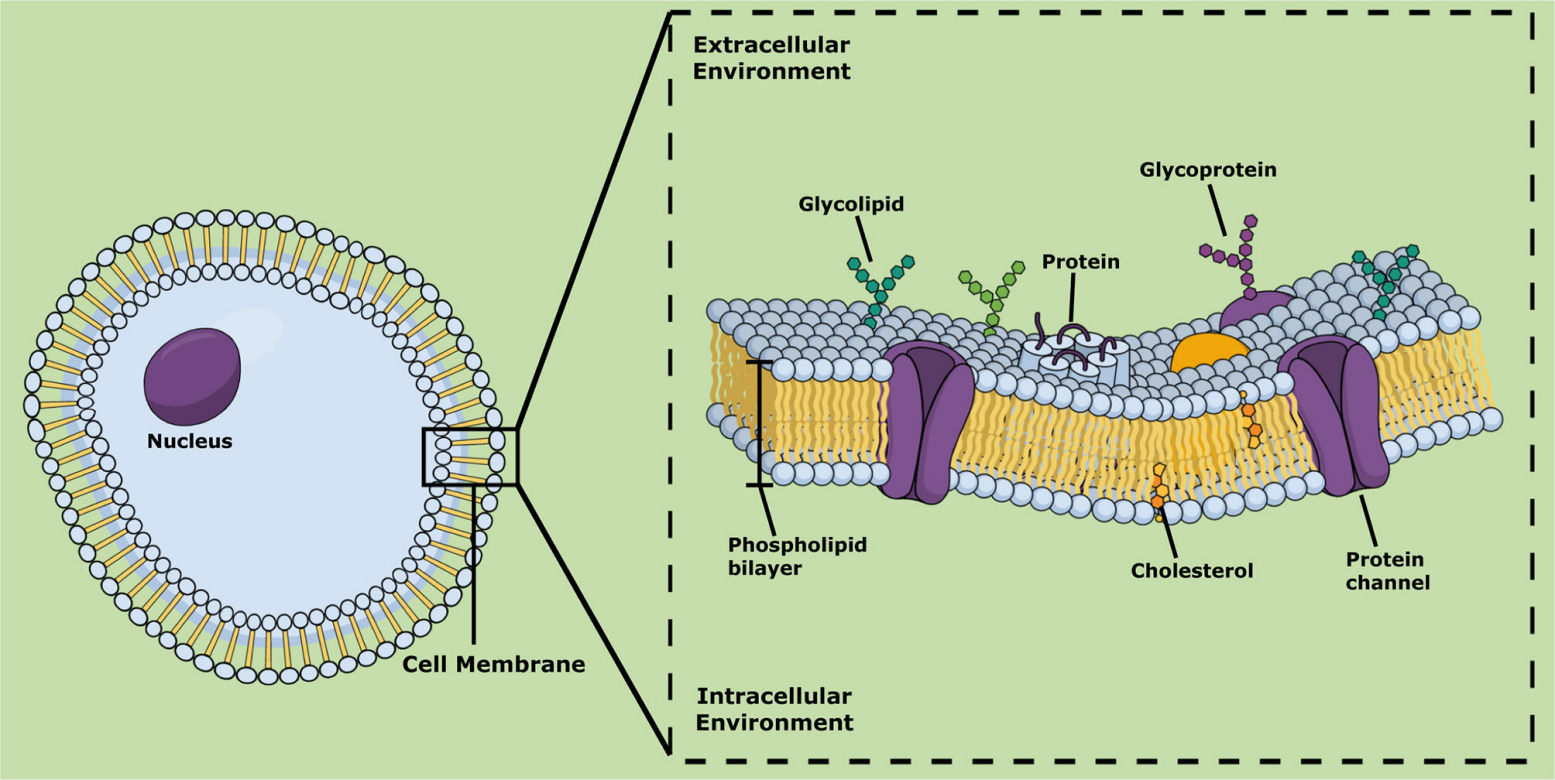
Schematic representation of cell membrane showing its main chemical lipidic and protein components.

The cell membrane (also known as cytoplasmic membrane) separates the cell from the exterior environment (Yang and Hinner, 2015). This biomembrane consists of a phospholipid bilayer that contains cholesterol between phospholipids that maintain their fluidity (Szlasa et al., 2020). Lipids of the cell membranes are highly diverse, and their structures vary in the extent of saturation of the fatty acyl chains. Three major classes of lipids can be distinguished in the cell membrane: sterols, phosphoglycerides, and sphingolipids (Harayama and Riezman, 2018) (**Figure 1**). Furthermore, the lipid compositions of the inner and outer monolayers are different, due to the different functions performed by the two faces (Guschina and Harwood, 2008; Harayama and Riezman, 2018). The cell membrane controls the passage of organic molecules and ions inside the cell, maintaining its homeostasis (Derakhshankhah and Jafari, 2018). The cell membrane contains several transmembrane, peripheral, and lipid-anchored proteins that perform a wide variety of molecular functions, including ion transportation, cell adhesion, cell signaling, and catalysis (Yang and Hinner, 2015).

**Figure 2:**
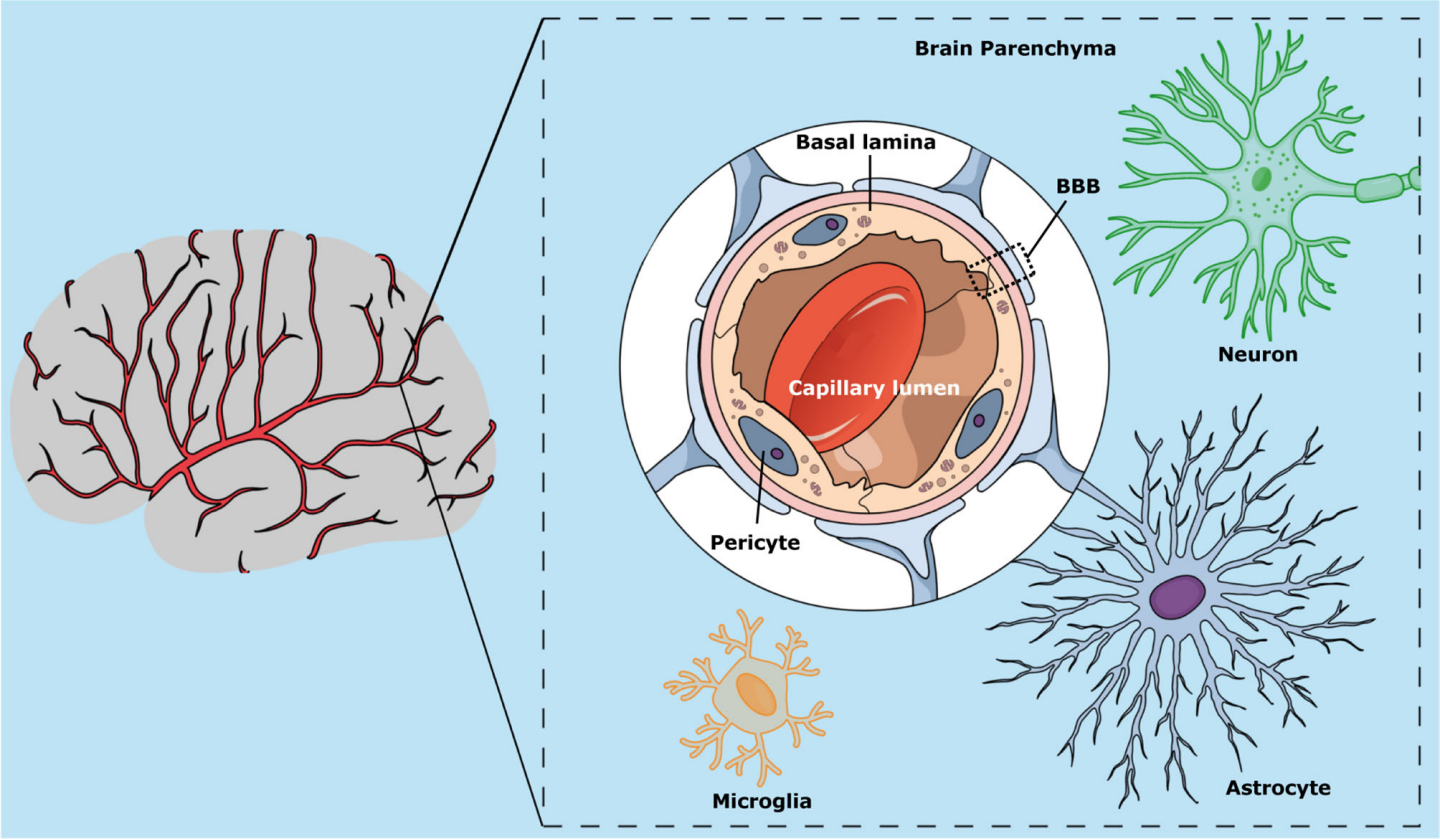
Schematic representation of the blood-brain barrier, showing its main cell components (pericytes, astrocytes, and endothelial cells) and localization in the brain capillary wall.

The BBB is a selective biomembrane that acts as a physical and chemical barrier of molecules of the central nervous system (CNS), controlling the homeostasis of the brain (Oller-Salvia et al., 2016). The BBB is mainly composed of endothelial cells present on the brain capillary walls that form tight junctions among adjacent cells. Other cell types present in the BBB include astrocytes and pericytes (**Figure 2**) (Daneman and Prat, 2015; Zaragozá, 2020). The BBB restricts the passage of pathogens and toxins while allowing the diffusion of some solutes present in the blood to the cerebrospinal fluid (Daneman and Prat, 2015; Zaragozá, 2020).

The development of molecules with high permeability into the biomembranes is one of the biggest obstacles faced by drug-oriented therapy strategies (Whitty et al., 2016; Sugita et al., 2021). Some bioactive molecules show high molecular weight and a strong hydrophilic nature which impair their entrance into their biological compartments of molecular action (Alex et al., 2011).

The determination of the physicochemical and structural parameters that govern the permeability of compounds into biomembranes remains an open research field of investigation and several studies have pointed out molecular properties that are relevant to predicting the permeability of these compounds (Daina and Zoete, 2016; Matsson et al., 2016; Matsson and Kihlberg, 2017; Rossi Sebastiano et al., 2018). Furthermore, some experimental methods have been applied to classify and predict the permeability of compounds, as well as to validate the predictive efficiency of the *in silico* models (Roy et al., 2019a; Sugita et al., 2021; Wadhwa et al., 2021; Radan et al., 2022).

In the present review, we discuss the main chemical parameters and experimental measurements involved in the determination of the permeability of molecules into the biomembranes and we correlate them with the main computational tools and approaches applied to investigate the peptides’ uptake. Additionally, we exhibit some biotechnological and pharmaceutical applications of biomembrane-penetrating peptides. Finally, we discuss some of the current limitations and perspectives of the development of these in silico approaches.

### Bioavailability of Compounds and Cell Membrane Permeability

The bioavailability of some compounds is intimately related to the efficiency of their penetration in biological membranes and their intrinsic solubility (Yang and Hinner, 2015). With regards to the oral bioavailability, for example, it has been mainly described as a function of the gastrointestinal absorption that occurs predominantly in the intestinal lumen (Bergström et al., 2016; Alqahtani et al., 2021). The microvilli of the cells present in the intestinal lumen are specialized membranes that show a high surface area that permits to the cells, a high absorption rate of nutrients and exogenous molecules, thus conferring the main absorption route of the gastrointestinal tract (Alqahtani et al., 2021). Based on this main mechanism of absorption, the gastrointestinal penetration of compounds has been computationally investigated through the analyses of the intrinsic compound solubility, the solubility in the aqueous phase, and cell membrane permeability (Bergström et al., 2016; Daina and Zoete, 2016). The intrinsic solubility of compounds is usually investigated by analysing the lipophilicity, aromaticity, and molecular flexibility (Delaney, 2004; Ottaviani et al., 2010; Ali et al., 2012).

### Chemical Properties Related to Cell Membrane Permeability

Physicochemical and structural properties related to permeability into cell membranes were initially determined by studies that investigated the bioavailability and solubility of compounds (Lovering et al., 2009; Lovering, 2013; Daina et al., 2014; Doak et al., 2014; Daina and Zoete, 2016; Matsson et al., 2016; Rossi Sebastiano et al., 2018). These molecular properties include the topological polar surface area (tPSA), partition coefficients between the lipid and aqueous phases calculated through logP and logD (pH 7.4), number of rotatable bonds (NRB), fraction of sp3-hybridized carbon atoms (Fsp3), molecular weight (MW), hydrogen bond acceptor (HBA), hydrogen bond donor (HBD), and number of aromatic rings (NAR) (Veber et al., 2002; Lovering et al., 2009; Lovering, 2013; Doak et al., 2014; Daina and Zoete, 2016; Matsson et al., 2016; de Oliveira et al., 2021).

Experimental findings have demonstrated that uptake into cell membrane is highly correlated with the partition coefficients between water and organic solvents (Arnott and Planey, 2012; Andrić and Héberger, 2015; Naylor et al., 2018). The lipophilicity, logP, and logD (pH 7.4) are the most widely applied parameters to investigate the intrinsic solubility of molecules that directly imply, as a result, their cell permeability (Doak et al., 2014; de Oliveira et al., 2021).

**Figure 3:**
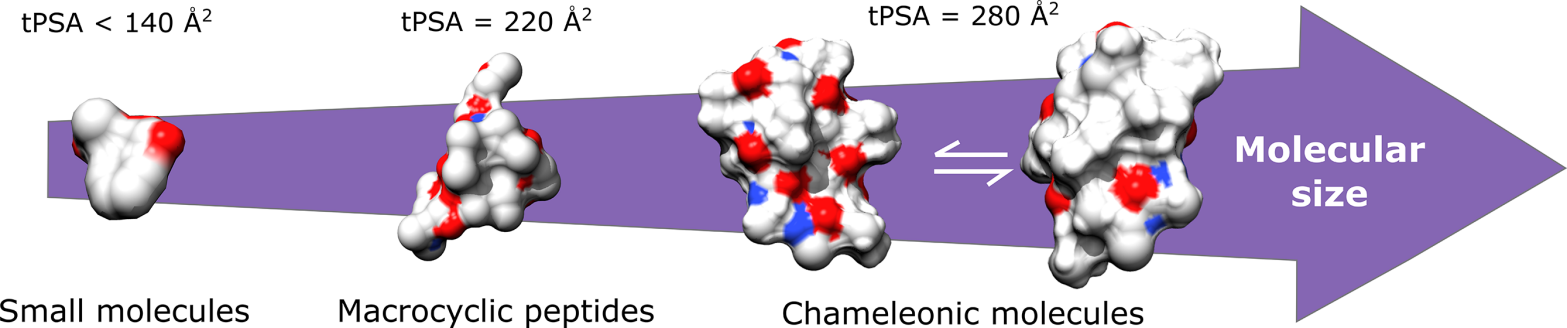
Acceptable tPSA values for the cell membrane permeability. Chameleonic molecules are able to change their conformation to expose polar groups in an aqueous phase, however hide them when translocating through the cell membranes.

Studies have demonstrated that the tPSA is correlated with the hydrogen bond pattern of the molecule in the aqueous phase. High values of tPSA are related to the complexation with the water molecules and with an increased molecular volume, which impairs the membrane permeability (Bergström et al., 2016). The penetration of compounds across cell membranes is typically limited when tPSA exceeds 140 Å^2^. However, values higher than this limit are usually acceptable for macrocyclic peptides (tPSA = 220 Å^2^) and peptides with chameleonic properties (tPSA = 280 Å^2^) (Matsson and Kihlberg, 2017; Rossi Sebastiano et al., 2018). Chameleonic properties refer to the ability of some peptides to change their conformation to expose polar groups in an aqueous phase, hiding them when translocating through the cell membranes (See **Figure 3**) (Whitty et al., 2016).

Molecular parameters related to the flexibility and complexity of compounds, such as NRB, Fsp3, and MW have been indicated to influence the translocation of molecules in the mobile aqueous phase due to the reduced entropic environment (Veber et al., 2002; Lovering et al., 2009; Lovering, 2013). The NRB evaluate the flexibility of molecules. High flexible structures can form intrachain hydrogen bond interactions, thus adaptively reducing their polarity surface and improving their permeability into the cell membrane (Kuhn et al., 2010). The Fsp3 is related to the solubility of molecules in the aqueous phase and melting point (Lovering et al., 2009). The NAR has been also pointed to as relevant structural parameter related to the compound lipophilicity and flexibility. Increases in its value usually result in a significant increase in the logP in the molecule structure (Ritchie and Macdonald, 2009).

### Chemical Properties Related to BBB Permeability

With regards to the chemical parameters that govern the permeability of molecules into BBB, different evidences have pointed out an interesting correlation between electrostatic interactions and polar surface area with the experimental values applied to the determination of BBB permeability (Shityakov et al., 2013; Thai et al., 2020). The lipophilicity has also a positive correlation with the BBB permeability (Hosoya et al., 2010; Carpenter et al., 2014). In contrast, the presence of a large number of hydrogen bonds is associated with experimentally low permeability of the BBB (Thai et al., 2020).

Dipole potential is another crucial factor involved with the passive penetration mechanism of the lipid bilayer. Some charged molecules are able to modify the membrane dipole potential by forming electrostatic interactions with the phosphatidylcholine heads of the lipidic bilayer, leading to attractions or repulsions (Cattelotte et al., 2009; Wang et al., 2019).

Recently, a study performed a statistical-based analysis of the structural and physicochemical properties shared by molecules that exhibit BBB penetration and indicated that nine molecular descriptors showed highly significant χ2 distribution: logD, logP, nitrogen and oxygen count, nitrogen count, oxygen count, ionization state, hydrogen bond acceptors, hydrogen bond donors, and polar surface area (Dichiara et al., 2020). Interestingly, another study demonstrated that *in silico* models applied in the prediction of BBB penetration that considered tPSA, HBD, and HBA values have shown superior performance to predict this class of peptides when compared with other classes of structural descriptors. These results demonstrate an essential role of polar interactions in the BBB penetration (Zhao et al., 2007). Studies have also demonstrated that molecular descriptors based on the solvation free energy calculated through 3D-RISM-KH theory, such as highest occupied molecular orbital (HOMO), dipole moment, HBA, and HBD play an important role in the BBB partitioning and could satisfactorily model the permeability of molecules into BBB (Lombardo et al., 1996; Roy et al., 2019a; Roy et al., 2019b).

### Experimental Parameters Related to the Permeability Into Biomembranes

The permeability into the biomembranes has been determined by different experimental methods that include artificial membrane-based assays, such as parallel artificial membrane permeability assay (PAMPA) - applied for cell membranes – and blood-brain barrier specific PAMPA (BBB-PAMPA), which contains adjusting in the lipid composition of the original artificial membrane – applied for the BBB; as well as cell-based assays, such as Caco-2 assay – applied for cell membranes, and bovine brain microvessel endothelial cells (BBMEC) assay – applied for BBB (He et al., 2020; Radan et al., 2022).

Currently, there are two main experimental parameters used to evaluate BBB permeability: logBB, and logPS. The logBB is the blood-brain partitioning data and it represents the concentration of the molecule in the brain divided by the concentration in the blood (Geldenhuys et al., 2015), according to Equation 1:


(1) $$\text{logBB }=\text{ log}\frac{{\left[molecule\right]}_{brain}}{{\left[molecule\right]}_{blood}\;}$$

The logPS represents the permeability surface-area product and this parameter is usually measured using the perfusion method. The logPS can be calculated using the Renkin-Crone equation (Eq 2):


(2) $$logPS=log-F\;\text{ln}\left(1-\frac{K_{in}}{F}\right)$$

Where F is the perfusion flow rate, and *K*_in_ is the unidirectional transfer constant. The *K*_in_ is equal to (Q_br_/C_pf_)/T, where Q_br_ is the concentration of the molecule in the brain, C_pf_ is the concentration of the molecule in the perfusion fluid, and T represents the perfusion time.

Other experimental parameters, such as effective permeability coefficient (P_eff_), efflux ratio, membrane retention, and logP are determined to evaluate both cell membrane and BBB permeability, and in general, apply cutoff values that indicate the efficiency of membrane permeation (He et al., 2020; Wadhwa et al., 2021).

Some computational models are based on these *in vivo* and *in vitro* experimental data or use them as comparative parameters to evaluate the prediction efficiency of the permeability into the biomembranes (Carpenter et al., 2014; Thai et al., 2020; Wadhwa et al., 2021). However, the methods applied to obtain these molecular properties are time-consuming and technically challenging; and may also display a slightly different trend when compared to each other, depending on the compared experimental procedure or protocol (Martin, 2004; Vastag and Keseru, 2009; Bagchi et al., 2019).

It is important to note that some *in silico* predictive models, such as cheminformatic filters and molecular dynamics (MD) simulations, usually assume passive diffusion as the main transport route of molecules through biomembranes. Thus, the active transport pathways, such as active influx transport, receptor-mediated transcytosis, and carrier-mediated transcytosis are usually not considered in the development of some models, due to the complex membrane protein binding processes which involve stereoselectivity (Bagchi et al., 2019).

## Peptides with Penetration into Biomembranes: Cell-penetrating Peptides and Blood-Brain Barrier-penetrating Peptides

Peptides comprise a versatile class of biomolecules with high biocompatibility that present unique pharmacokinetic and pharmacodynamic properties (Kumar et al., 2018a; Capecchi and Reymond, 2021). Some classes of peptides, such as cell-penetrating peptides (CPPs) and blood-brain barrier-penetrating peptides (B3PPs) have been explored as carrier systems of bioactive molecules, such as gene constructs (Davoodi et al., 2019), small interfering RNAs (siRNAs) (Pärnaste et al., 2017; Tai and Gao, 2017; Tuttolomondo et al., 2017), and drugs (Ramsey and Flynn, 2015; Park et al., 2019).

CPPs can naturally cross cell membranes without the intermediation of molecular receptors (Koren and Torchilin, 2012). CPPs possess a wide range of biological activities (Farkhani et al., 2014), such as antifungal (Budagavi et al., 2018), antibacterial (John et al., 2019; Lee et al., 2019), neuroprotective (Baig et al., 2018a; Jiang et al., 2021), and antiviral (Keogan et al., 2012; Zhang et al., 2020) activities. In addition, this class of peptides has been also explored as delivery systems of drugs, small interfering RNA (siRNA), and gene constructs (Patel et al., 2019; Silva et al., 2019; Klabenkova et al., 2021). CPPs have been described sharing different structural and physicochemical properties: they are often amphipathic or cationic (positive charge at physiological pH), show sequence length between 5 and 40 amino acids, their structures are soluble in water and partially hydrophobic, and their amino acid sequence are rich in lysine and arginine residues (Milletti, 2012).

Several penetration mechanisms through cell membranes have also been described for CPP structures, such as translocation by passive diffusion, pore formation, translocation across endosomal membrane, and endocytosis (Gestin et al., 2017). Based on the physicochemical properties, the CPPs are classified into three categories: (1) amphipathic (*e.g.:* MAP and Pep1), (2) cationic (*e.g.:* Tat and Arg_9_), and (3) hydrophobic (*e.g*.: TP2) (Milletti, 2012). Amphipathic CPPs can also be divided into four subcategories based on their hydrophilic and hydrophobic domains, as well as their topology: (1.1) primary amphipathic that include peptides defined by their hydrophobic domains, (1.2) secondary amphipathic (or amphipathic α-helical) that forms α-helices with one hydrophilic face and one hydrophobic face, (1.3) amphipathic β-sheet that have a hydrophilic stretch and a hydrophobic stretch, and (1.4) proline-rich amphipathic peptides, that form polyproline II (PPII) structures (Milletti, 2012; Reid et al., 2019).

B3PPs, also known as brain-penetrating peptides or BBB shuttle peptides, represent oligopeptide chains with permeability into the BBB that represent interesting biotechnological applications due to their favoring the increase in the brain uptake of large molecular cargoes in a non-selective way (Geldenhuys et al., 2015; Oller-Salvia et al., 2016; Díaz-Perlas et al., 2018). These peptides have been extensively investigated aiming the development of new chemotherapeutic compounds due to their antiviral (Jackman et al., 2018), anticancer (Chen et al., 2019), and neuroprotective activities (Meloni et al., 2014).

Some B3PPs belong to the CPP family, such as SynB3, Tat 47–57, and pVEC. However, cell-penetrating properties of peptides do not necessarily imply the ability to penetrate the BBB (Stalmans et al., 2015). Studies have demonstrated that both classes of peptides comprise different structural and physicochemical properties (de Oliveira et al., 2021; Zou, 2021). The diffusing of peptides across the BBB have been better correlated with hydrogen bonding and water desolvation than logP (Chikhale et al., 1994). It has also been demonstrated that B3PPs contain a differential content of lysine, tyrosine, glycine, and arginine residues in their sequences when compared with other classes of peptides (Kumar et al., 2021b). Based on the physicochemical criterion, three families of BBB-penetrating peptides were defined: diketopiperazines (Teixidó et al., 2007), N-methylphenylalanines (Malakoutikhah et al., 2010), and phenylprolines (Arranz-Gibert et al., 2015). Some BBB-penetrating peptides have also been described with different crossing mechanisms of the membrane, such as receptor-independent (e.g.: adsorptive-mediated transcytosis), and receptor-dependent mechanisms (e.g.: receptor-mediated transcytosis) (Lu, 2012). These peptides have been applied as a strategy to cross BBB by endogenous transcytosis mechanism and invade brain parenchyma allowing bioactive molecules to reach the CNS (Lee and Jayant, 2019; Zhou et al., 2021).

## Computational Approaches Applied in the Prediction of Biomembrane-penetrating Peptides

Structure- and sequence-based information of peptides, as well as atomistic biophysical models, have been used in computer-assisted discovery strategies to identify new structures with penetration in biological membranes (Carpenter et al., 2014; Santana et al., 2021), as well as in order to explore their molecular mechanism of penetration (Thai et al., 2020). Computational strategies applied to predict the permeability of peptides and other small molecules into biomembranes include cheminformatic filters (Jeffrey and Summerfield, 2010; Wager et al., 2010), molecular dynamics simulations (Carpenter et al., 2014; Wang et al., 2019), artificial intelligence algorithms (Schaduangrat et al., 2019; Alsenan et al., 2020; Dai et al., 2021; de Oliveira et al., 2021), and statistical models (Daina and Zoete, 2016; Daina et al., 2017). The computational prediction of biomembrane-penetrating peptides has gained continually attention of research groups due to the low-cost approaches when compared to experimental methods that use solely experimental assays (Derakhshankhah and Jafari, 2018; Qiang et al., 2018; Kumar et al., 2021a; de Oliveira et al., 2021).

These computational methods usually apply molecular data calculated computationally to predict the passive permeability of peptides into biomembranes, and validate their results using experimental data (Rezai et al., 2006; Dai et al., 2021; de Oliveira et al., 2021; Kumar et al., 2021a; Shaker et al., 2021; Sugita et al., 2021).

### Cheminformatic Filters

Cheminformatic filters were one of the first *in silico* models to predict the permeability of molecules into the biomembranes (Lipinski et al., 1997; Doak et al., 2014). These computational models assume that the passive penetration of molecules into these membranes is influenced predominantly by a set of physicochemical and structural properties of compounds that regulate their structural flexibility, solubility, and biophysical interactions with the biomembranes (Wang et al., 2007; Doak et al., 2014; Yang and Hinner, 2015). These filters are based on numerical intervals of a set of molecular properties that can be obtained using *in silico* calculations. These properties represent a variation of the first reported ‘Lipinski’ rule of five (RO5) and Veber rules (Wager et al., 2010) that analyze the following molecular descriptors: logP, RTB, tPSA, HBA, HBD, and MW (Doak et al., 2014; Matsson et al., 2016; de Oliveira et al., 2021; Digiesi et al., 2021).

**Cheminformatic filters applied in CPPs prediction:** The CPPs has been described, at least in part, using the conventional filters applied to test the bioavailability and drug-likeness of molecules, such as Lipinski and Veber (Lipinski et al., 1997; Veber et al., 2002; Muegge and Mukherjee, 2016). However, it is well known that several CPP structures cannot be adequately predicted using these filters due to their unique chemical space, the existence of chameleonic properties, as well as the presence of diverse molecular mechanisms of cell membrane penetration that include phagocytosis and pore formation (Díaz-Eufracio et al., 2018; de Oliveira et al., 2021). The beyond the rule of five (bRO5) filter is most suitable to analyze some classes of compounds, such as cyclic peptides that are located beyond the chemical limits determined by conventional filters (Matsson et al., 2016; Rossi Sebastiano et al., 2018). **Table 1** describes some cheminformatic filters applied in predicting of bioavailability of drugs and peptides (Lipinski et al., 1997; Veber et al., 2002; Doak et al., 2014; Díaz-Eufracio et al., 2018) and compares them with the previously reported chemical space of CPPs (de Oliveira et al., 2021).

**Table 1:** Comparison between the chemical spaces and cheminformatic filters of peptides and commercial drugs with bioavailability.

Molecular properties	Oral drugs			Peptides		
	Lipinski et al. (1997)	Veber et al. (2002)	Doak et al. (2014)	Santos et al. (2016) ^*^	Diaz-Eufracio et al. (2018) ^**^	de Oliveira et al. (2021) ^***^
**MW**	≤ 500	–	≤ 1,000	≤ 700	27.03 ≤MW ≤5,036.65	331.48 ≤ MW≤ 3,750.51
**logP**	≤ 5	–	-2 ≤ cLogP ≤ 10	≤ 7.5	-17.87 ≤ cLogP ≤ 39.89	-42.12 ≤ cLogP ≤ 2.97
**tPSA**	–	≤ 14	≤ 250	≤ 200	≤ 2,064.83	101.29 ≤ tPSA ≤ 1,782.83
**Fsp^3^**	–	–	–	≤ 0.55	–	0.37 ≤ Fsp^3^ ≤ 0.84
**NRB**	–	≤ 10	≤ 20	≤ 20	≤ 209	9 ≤ NRB ≤ 137
**HBD**	≤ 5	*-*	≤ 6	≤ 5	≤ 76	4 ≤ HBD ≤ 69
**HBA**	≤10	*-*	≤ 15	≤ 10	≤ 71	5 ≤ HBA ≤ 55
**NAR**	–	–	–	–	–	≤ 10

*Investigated oral available peptides; **Investigated the linear and cyclic pentapeptides; ***Investigated CPP structures and described their chemical space.

Table edited from de Oliveira et al. (2021).

**Cheminformatic filters applied in B3PPs prediction**: The central nervous system multiparameter optimization (CNS MPO) filter is the most used cheminformatic model to evaluate the permeability of compounds through the BBB and it was built using a set of commercially available CNS drugs (119 compounds) and CNS candidates (108 compounds) and tested using a large set of proprietary compounds (11,303 compounds) (Wager et al., 2010). The CNS MPO uses six molecular parameters: HBD, logP, pKa, logD (pH = 7.4), MW, and tPSA that result in a 6-point scale. The CNS MPO has been widely implemented to evaluate the permeability of compounds into the BBB (Rankovic, 2017; Urbina et al., 2021). Similarly, Lagorce et al. (2015) developed a molecular filter that bases its screening on the statistical analysis of molecular properties obtained from small molecules, using the following cutoff values: MW (135 - 582), logP (-0.2 to 6.1), HBA (≤ 5), HBD (≤ 3), and tPSA (3 – 118) (Lagorce et al., 2015).

### Artificial Intelligence Algorithms

Artificial intelligence (AI) is a field of study that develops algorithms for machines to learn patterns from a set of data to find solutions for real-world problems based on a cognitive behavior associated with the human brain (Hessler and Baringhaus, 2018). Currently, several AI models have been developed to solve different types of biological and chemical problems (Sato et al., 2010; Dimitri and Lió, 2017; Yang et al., 2018; Nocedo-Mena et al., 2019; Shoombuatong et al., 2019; Zoffmann et al., 2019; Kong et al., 2020; Miao et al., 2021; Oršolić et al., 2021). Machine learning (ML) is one field of artificial intelligence that has considerably increased in the last decades (Garg and Mago, 2021). It represents the science that develops and studies algorithms able to learn patterns from data (training) and return information from new ones (testing) (James et al., 2013; Saldívar-González et al., 2022).

**Figure 4:**
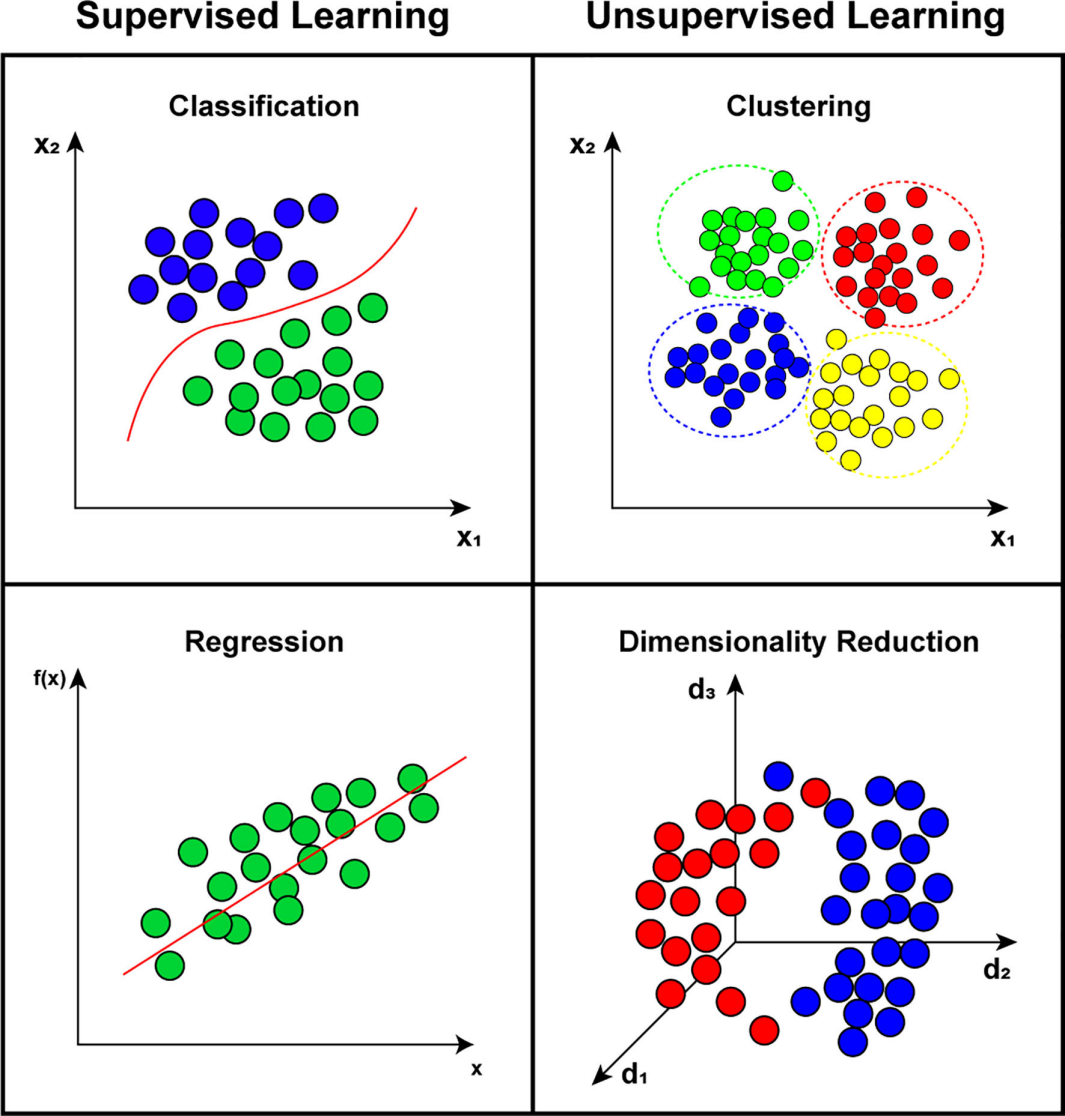
Representation of main categories on machine learning state-of-art, divided between supervised learning, such as classification and regression problems; and unsupervised learning that includes clustering and dimensionality reduction problems.

Several ML techniques have been proposed to solve different computational problems, including classification, time series regression, natural language processing, optimization, and dimensionality reduction. For example, classification and regression problems can be solved with artificial neural network (ANN), deep learning (DL), k-nearest neighbors (k-NN), support vector machine (SVM), decision tree (DT), and random forest (RF) (Balaji et al., 2021). Clustering problems can be treated using k-means, hierarchical cluster analysis (HCA), and DBSCAN. Visualization and dimensionality reduction problems can be solved using principal component analysis (PCA), locally-linear embedding (LLE), and t-distributed stochastic neighbor embedding (t-SNE) (Yau et al., 2019; Roohi et al., 2020). **Figure 4** illustrates the main categories of ML science divided into two categories: (1) supervised learning that consists of algorithms that need labels or information about the output of a time series to be trained; and (2) unsupervised learning that corresponds to techniques that do not use any previous information to be trained.

The prediction of biomembrane-penetrating peptides has been an interest of the biotechnological and pharmaceutical industries due to the development of new therapeutic compounds and carrier systems. ML algorithms have been a valuable tool to predict the pharmacokinetic properties of peptides with reduced costs and time. Different studies have been reported success in the prediction of biomembrane-penetrating peptides (Pandey et al., 2018; Fu et al., 2020; de Oliveira et al., 2021; Kumar et al., 2021a). ML algorithms also have been widely applied in the virtual screening of large compound libraries belonging to different chemical classes (Bhadra et al., 2018; Galúcio et al., 2019; Shoombuatong et al., 2019; Oršolić et al., 2021; Santana et al., 2021).

**ML-based models applied for CPPs prediction:** Regarding the prediction of CPPs, Pandey et al. (2018) proposed an ML framework named KELM-CPPpred that applies a kernelized extreme learning machine (ELM) and uses as molecular descriptor the amino acid composition of the peptides sequences to differentiate CPP from non-CPP sequences. Similarly, Qiang et al. (2018) developed a tool named CPPred-FL that applies 45 trained RF models using 19 descriptors related to amino acid composition, specific-position information, and physicochemical properties to predict CPPs. Fu et al. (2019) developed an ML algorithm using SVM with an RBF kernel to predict CPPs using as feature composition the amino acid composition of the sequences.

**Figure 5:**
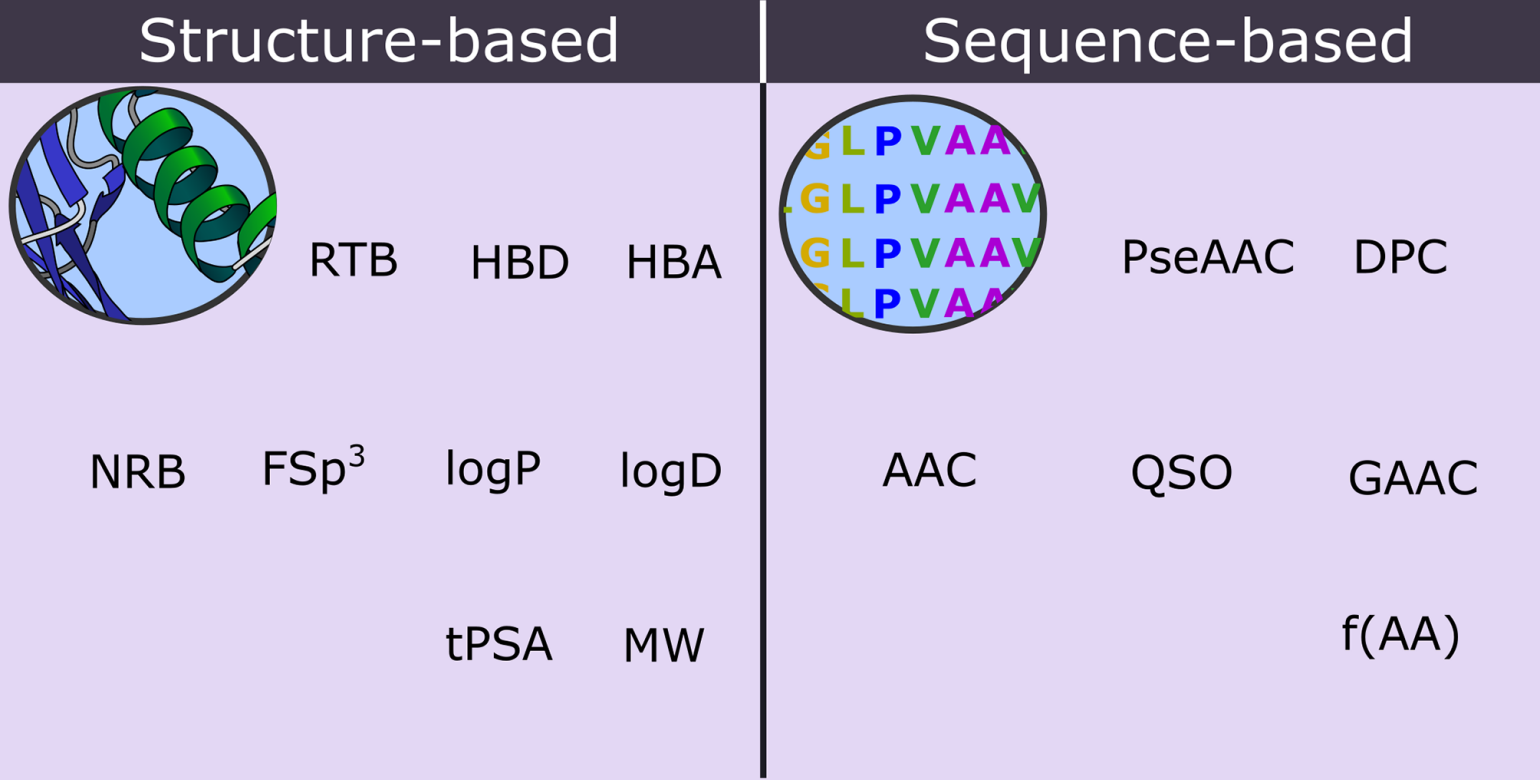
Structure- and sequence-based molecular descriptors that are applied in the prediction of biomembrane penetrating peptides.

It has been demonstrated that ML algorithms that use an optimized combination of sequence- and structure-based descriptors have a superior accuracy when compared with those that use only sequence- or only structure-based descriptors (Manavalan et al., 2018; de Oliveira et al., 2021). Sequence-based descriptors of peptides are associated with information calculated from the primary structure of the peptide. Examples of these descriptors include the fraction of a specific amino acid (f[AA]), amino acid composition (AAC), pseudo-amino acid composition (PseAAC), dipeptide composition (DPC), quasi-sequence order (QSO), and grouped amino acid composition (GAAC) (Chen et al., 2016; Wei et al., 2017; Pandey et al., 2018). In contrast, the structure-based descriptors are associated with structural and physicochemical properties, such as NAR, NRB, Fsp3, logP, MW, tPSA, etc (de Oliveira et al., 2021). Some hybrid descriptors, such as the ‘composition, transition, and distribution (CTD)’, include attributes obtained directly from the polypeptide chain, including the secondary structure, solvent accessibility, normalized van der Waals volume, polarity, hydrophobicity, polarizability, and charge (Govindan and Nair, 2011). **Figure 5** shows an overview of the two classes of structure- and sequence-based descriptors of peptides applied in the prediction models.

Recently, Manavalan et al. (2018) proposed a ML framework based on the physicochemical properties and amino acid sequence composition of peptides to predict CPPs and non-CPPs using a combination of different ML algorithms that include SVM, RF, ERT, and K-NN (Manavalan et al., 2018). Molecular fingerprints associated with structure and sequence-based have also been applied in some ML algorithms to predict CPP structures. Based on these descriptors, Kumar et al. proposed the CellPPD-Mod, a computational tool that uses RF to differentiate CPPs from non-CPPs with lengths up to 25 residues using molecular fingerprints, amino acid composition descriptors, and 48 two-dimensional (2D)/three-dimensional (3D) molecular features (Kumar et al., 2018b). Similarly, de Oliveira et al. (2021) proposed the BChemRF-CPPred, a ML framework based on ANN, SVM, and Gaussian process classifier (GPC) that uses as input data an optimized combination of sequence-based and structure-based features of peptides (de Oliveira et al., 2021).

**ML-based models applied in B3PPs prediction:** BBB-penetrating peptides have also been predicted using ML algorithms (Dai et al., 2021; Liu et al., 2021). Dai et al. (2021) proposed the BBBpred, a computational tool that uses sequence-based features and involves different ML algorithms to performs the B3PP prediction, such as extremely random tree (ERT), eXtreme gradient boosting (XGB), KNN, multilayer perceptron (MLP), RF, SVM, and logistic regression (LR) (Dai et al., 2021). Similarly, Kumar et al. proposed the B3Pred, a computational tool to evaluate the prediction of the permeability of three datasets of natural and synthetic peptides that uses as molecular descriptors some structure-based properties combined with multiple ML algorithms, such as DT, RF, LR, KNN, Gaussian Naive Bayes (GNB), XGB, and SVM (Kumar et al., 2021a).

Zou (2021) proposed using SVM to identify B3PPs employing physicochemical properties of the peptides. The authors used Pearson’s correlation coefficient and maximal information coefficient to extract useful information to predict the permeability, which was integrated by a similarity network fusion algorithm (Zou, 2021). Recently, He et al. (2021) proposed a mutual information maximization meta-learning (MIMML) algorithm, a novel deep meta-learning method that predicts bioactive peptides using sequence-based descriptors and applies mutual information maximization and convolution kernel. This ML framework was developed to predict different classes of bioactive peptides, such as anti-angiogenic, anti-bacterial, anti-tubercular, anti-fungal peptides. Specifically, for B3PPs, the MIMML outperformed when compared to its counterpart algorithms (He et al., 2021).

Although some ML-based tools have been developed exclusively to predict B3PPs using sequence and structure-based descriptors obtained from peptides, other studies explored these algorithms to classify small molecules according to their penetration through the BBB. Recently, Shaker et al. (2021) used Light Gradient Boosting Machine (LightGBM) algorithm to predict the penetration of small molecules across the BBB using as input data 2,432 1D/2D descriptors calculated using the Dragon program (Shaker et al., 2021). Similarly, Liu et al. (2021) used SVM, RF, XGB, and ensemble models to predict the uptake of small molecules by the BBB using as feature nine molecular fingerprints (EState, MACCS, PubChem, FP4, KR, AP2D, FP4C, KRC, and APC2D) (Liu et al., 2021). In another study, Urbina et al. (2021) proposed a Bayesian ML model to predict the penetration of active and inactive compounds using extended connectivity fingerprint descriptors and compared the proposed method with two versions of CNS MPO algorithms termed Pf-MPO.v1 and Pf-MPO.v2 (Urbina et al., 2021).

### Quantitative Structure-Property Relationships

Quantitative structure-property relationships (QSPRs) are statistical methods based on regression data analyses that have been widely applied in Computational Chemistry to perform bioprospection and develop new bioactive compounds (Melo-Filho et al., 2016; Gomes et al., 2017; Rajathei et al., 2020; Tutumlu et al., 2020; Al-Attraqchi and Venugopala, 2021). The QSPR represents mathematical models that aim to create correlations between the biological property (e.g.: toxicity, penetration in biomembranes, etc) and a set of descriptors obtained from the analyzed compounds (Neves et al., 2018; Toropov and Toropova, 2020). Among the molecular descriptors applied in QSPR analyses we can cite physicochemical (logP, MW, pKa, etc), structural (NAR, NRB, etc), atomic (number of O, N, etc) properties, as well as experimental data (logBB, logPS, etc). However, QSPR methods depend on the existence of experimental information previously deposited in online databases or on the performing of experimental tests.

Several criteria have been chosen to evaluate the quality of those models, including the total number of compounds applied in the training dataset, results of the correlation coefficients; and standard error estimation (Neves et al., 2018). The QSPR models have been applied to evaluate the penetration of different classes of compounds into the biomembranes, including the cell membrane (Dowaidar et al., 2017) and the BBB (Zhang et al., 2008; Fan et al., 2010; Bujak et al., 2015). The QSPR models that predict the penetration of compounds into BBB use some experimental parameters applied to evaluate the penetration, such as logBB and logPS (Nikolic et al., 2013). QSPR models, clustering, and correlation studies that use these experimental data to evaluate the BBB permeation demonstrated a success rate for different classes of compounds (Nikolic et al., 2013; Bujak et al., 2015). Similarly, QSPR studies have also been combined with ML algorithms to predict the permeability of compounds into the BBB (Zhang et al., 2017).

### Molecular Dynamics Simulations

Molecular dynamics (MD) simulations are physics-based computational methods that implement iterative algorithms to calculate velocities, atomic positions, and acceleration of a set of molecules over time, thus providing a detailed atomistic analysis of their structure, trajectories, interactions, and conformational changes (Karplus and McCammon, 2002; Câmara and Horjales, 2018; Reid et al., 2019). Two approaches have been widely applied in MD simulations to describe the dynamic behavior of molecules: molecular mechanics (MM) and quantum mechanics (QM). MD simulations that apply the classical MM approach describes the atomic movement using Newtonian equations, thus allowing us to investigate the trajectory of biomolecules using reasonable computational efforts when compared with the quantum approaches. However, these simulations are inadequate to analyze chemical reactions, electron transfer, and transition states (Field, 2007). In contrast, MD methods that apply QM solely or a hybrid approach (QM/MM) have been useful to describe these molecular mechanisms (Ahmadi et al., 2018).

Despite cheminformatic filters, QSPR models, and ML algorithms have been widely applied in the high-throughput screening of new biomembrane-penetrating molecules, they do not offer a detailed analysis of the molecular mechanism of penetration and the molecular interactions that occur between peptide and lipid bilayers (Lee et al., 2016; Reid et al., 2019). Explicit-solvent MD simulations are useful to predict the biomembrane-penetrating peptides, to describe their molecular mechanism of passive penetration, and to assess accurately the energetic and structural preferences of the peptide structures in the aqueous solutions (Dunkin et al., 2011; Horn et al., 2013; He et al., 2015; Mukherjee et al., 2017). Some MD packages, such as Chemistry at HARvard Macromolecular Mechanics (CHARMM) (Brooks et al., 2009), AMBER (Salomon-Ferrer et al., 2013), and GROningen MAchine for Chemical Simulations (GROMACS) (Van Der Spoel et al., 2005) contains useful molecular force fields to describe peptides-bilayer lipid systems, such as CHARMM (Huang et al., 2017), AMBERFF (Maier et al., 2015), and GROMOS (Reif et al., 2012), respectively. In addition, some coarse-grained force fields, such as MARTINI (Marrink et al., 2007) and SIRAH (Darré et al., 2015) have been developed to characterize these systems increasing the accessible simulation time. The choice of the most adequate force field to describe the peptide penetration depends on the analyzed system and the calculated properties, thus different results could be reached for independent simulations using the same protocol (Piggot et al., 2017).

**Figure 6:**
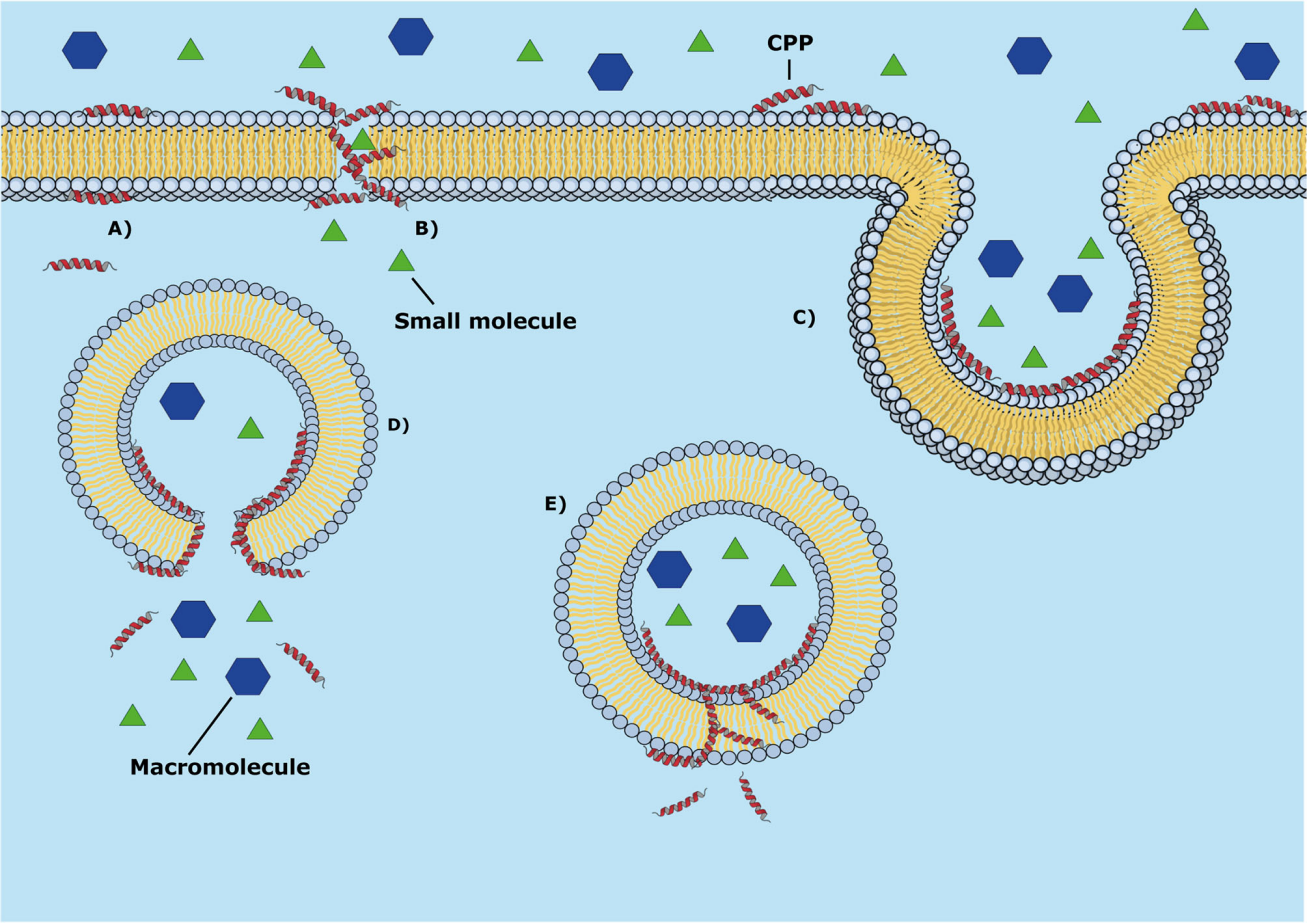
Mechanisms of passive penetration of CPPs into the cell membrane (energy-independent mechanisms). **(A)** Passive diffusion (spontaneous translocation), **(B)** Peptide aggregation with pore formation, **(C)** endocytosis. The panels **(D)** and **(E)** represent some subsequent molecular events in the cell: **(D)** endosomal membrane lysis and **(E)** translocation through the cell membrane.

With regards to the CPPs, these peptides possess a wide range of molecular mechanisms of cell membrane penetration that include passive (energy-independent) and active (energy-dependent) translocation (**Figure 6**). The energy-dependent mechanisms include passive penetration, endocytosis, and pore formation. MD simulations had been applied to elucidate some passive mechanisms of penetration in the cell using lipid bilayer models of cell membrane (Horn et al., 2013; Di Pisa et al., 2015; Mukherjee et al., 2017), and membrane-mimetic environments (Timmons and Hewage, 2021).

It is important to note that due to the high demand of computing resource required by the MD simulations to observe some molecular events, sometimes, it is not possible to assess, in the time scale applied in the simulation the complete translocation of peptides through the membrane. Therefore, some aspects involved with peptide penetration, such as the formation of intermolecular interactions, peptide aggregation, peptide orientation, and the influence of solved accessible surface could be useful to conjecture the possible molecular mechanism of penetration (Dunkin et al., 2011; Horn et al., 2013). In addition, the prediction of permeability of large molecules may be particularly prone to inaccuracy due to insufficient conformational sampling during the simulations (Sugita et al., 2021).

The passive penetration of small molecules into the cell membranes has been studied using solubility-diffusivity models, and the potential of mean force (PMF) is a critical component applied in the study of membrane permeability using MD simulations (Bennion et al., 2017; Venable et al., 2019; Wadhwa et al., 2021). To obtain a satisfactory sampling of the transition states of the investigated molecules, several techniques have been developed which include adaptive biasing force (Rodriguez-Gomez et al., 2004), replica-exchange umbrella sampling (Torrie and Valleau, 1977), metadynamics (Laio and Parrinello, 2002), and the Wang−Landau algorithm (Wang and Landau, 2001). These sampling techniques are useful to obtain the PMF of the molecule passing across membranes.

The PMF represented by *W*(z), as well as the permeability (*P*), the local diffusivity coefficient, *D*(z) and the resistivity (*R*) are mathematically related according to Equation 3:


(3) $$R=\frac{1}{P}\int_{z1}^{z2}\frac{\text{exp}\left[\frac{1}{\text{kBT}}W\left(z\right)\right]}{D\left(z\right)}dz$$

Where z is a collective variable (CV) that describes the relative position of the molecule along the transmembrane axis, and z1 and z2 represent points along this axis on opposing sides of the biomembrane (Lee et al., 2016; Venable et al., 2019). The 1/kBT corresponds to the inverse of the Boltzmann constant times the temperature.

The choice of CV is a crucial step for effectively obtaining the free-energy profile of the analyzed molecules. Recently, (Kabelka et al., 2021) developed a CV that includes a description of peptide insertion, local membrane deformation, and peptide internal degrees of freedom related to its charged groups which can be effective to calculate the free energy of peptide translocation and could be useful in the design of spontaneously translocating peptides.

## Biomembrane-penetrating Peptides Databases

Databases with experimentally validated information of biomembrane-penetrating peptides are a useful source to obtain structure- and sequence-based data for the development of computational models applied in the prediction of permeability of these structures. However, despite their valuable information regarding the penetration of the peptide in the biomembranes, the absence of data related to the penetration mechanism of some peptides, chameleonic properties, and shared pharmacokinetic properties of penetration in both membranes impairs, in some aspects, the development of more predictive computational models. In the present section, we exhibit the main public and online databases of CPPs and B3PPs.

**CPPsite 2.0:** This database is an updated version of CPPsite (Gautam et al., 2012) and contains experimentally validated CPP structures with high uptake efficiency into the cell membrane. CPPsite2 contains information on the primary, secondary, and tertiary structure of peptides, besides offers a similarity search engine that allows the users to search for peptides using the structure, sequence, and amino acid composition. There is also group-wise data browsing that allows users to retrieve data in different categories that include cyclic peptides, nucleic-acid delivering, synthetic and natural peptides, etc (Kardani and Bolhassani, 2021). CPPsite 2.0 is freely available at https://webs.iiitd.edu.in/raghava/cppsite.

**BrainPeps:** The database contains comprehensive data obtained from literature information about B3PPs, which include primary structure, sequence, physicochemical properties, and information related to the experimental method applied in the validation of the penetration (Van Dorpe et al., 2012). BrainPeps is freely available at https://brainpeps.ugent.be/.

**B3Pdb**: The database contains information of the primary structure of experimentally validated B3PPs. The information available in the database include the physicochemical properties, chemical modifications, and related references. The database also contains a similarity search engine and group-wise data browsing to assess the information using different peptide categories (Kumar et al., 2021b). The B3Pdb is freely available at https://webs.iiitd.edu.in/raghava/b3pdb/.

## Biotechnological and Pharmaceutical Applications of Biomembrane-penetrating Peptides

Since human insulin was produced by recombinant DNA technology and approved by the US Food and Drug Administration (US FDA), several advances in the biotechnological and pharmaceutical applications of natural and synthetic peptides have encouraged investments by the pharmaceutical industry (Baig et al., 2018b). The present section shows some pharmaceutical and biotechnological applications of biomembrane-penetrating peptides in the drug design and discovery, such as brain cargo delivery systems, antimicrobial, and anti-neurodegenerative disorders agents.

### Applications as Neuroprotective Agents

Neurodegenerative diseases are characterized by gradual and progressive degeneration of brain function and loss of neurons (Heemels, 2016; Ahmadian-Moghadam et al., 2020). Alzheimer’s disease (AD), Parkinson’s disease (PD), amyotrophic lateral sclerosis (ALS), Huntington’s disease (HD), and vascular dementia are examples of neurodegenerative disorders that affect millions of people worldwide, whose treatments have been insufficient due to the low number of approved drugs in clinical trials (Slanzi et al., 2020).

The BBB is the main obstacle to the effectiveness of treatments of neurodegenerative diseases due to its selective control of the penetration against some therapeutic agents that impairs them to achieve the CNS (Zhou et al., 2021). The B3PPs have become useful tools for the treatment of neurological disorders due to their ability to penetrate the membranes carrying neurotropic small molecules or to act as neuroprotective agents (Oller-Salvia et al., 2016).

In the last years, several natural and synthetic bioactive peptides have been tested in clinical trials to treat neurodegenerative diseases (Brinker and Spader, 2014; Baig et al., 2018a; Zhang et al., 2021). Some peptides affect directly biochemical processes in CNS which are involved in some neurological disorders, for instance, the Ziconotide has shown activity in blocking the increase of extracellular glutamate and intracellular overload of Ca^2+^, which are the main cause of neuronal injury during cerebral ischemia (Zieminska et al., 2006). Exenatide is a GLP-1R agonist extracted from venomous lizard *Heloderma suspectum* and it is approved for the treatment of diabetes type II. Some studies displayed that the administration of this peptide peripherally can cross the BBB in humans (Hunter and Hölscher, 2012; Athauda et al., 2017), besides exhibiting neuroprotective effects on several neurodegenerative models, mainly in PD (Athauda and Foltynie, 2016).

PhTx3-3 and PhTx3-4, peptides derived from spider *Phoneutria nigriventer* venom, have also shown activity as a blocker of Ca^2+^ channel, exocytosis in nerve endings, and glutamate release (Yang et al., 2019). Previous studies also showed that these peptides rescued the neurotransmission alterations observed in hippocampus CA1 and prevented neuronal cell death (Pinheiro et al., 2009).

Dysfunctions in mitochondrial metabolism have been reported as one factor related to the development of neurodegenerative diseases, such as AD and PD. Filichia et al. (2016) showed that the peptide P110 inhibited the DRP1, a protein essential to mitochondrial fission and dopaminergic neuronal death, preventing dopaminergic neurons in PD (Filichia et al., 2016). Ba-V peptides are obtained from *Bothrops atrox* snake venom and these structures have shown neuroprotective activity, acting against the mitochondrial permeability transition, a biochemical process that can trigger an intrinsic apoptotic pathway of neuronal cells that is related to PD and AD (Martins et al., 2010).

The B3PPs have also received attention from pharmaceutical industries due to their capacity to conjugate with drugs and other small molecules, implying the development of new and more effective therapeutic agents against neurodegenerative disorders. Among these peptides, we can cite transportan 10, angiopep-2, and SynB.

Transportan 10 is a peptide with a high penetration rate into the BBB. TP10-dopamine conjugate has been demonstrated to access brain parenchyma and possesses a high binding affinity to both dopamine D1 and D2 receptors, besides it has shown activity against PD in animal models (Rusiecka et al., 2019). TP10 conjugated with vancomycin also has shown the capacity to improve the bioavailability of this drug in the brain, 200-fold greater than free one (Ruczyński et al., 2019).

Angiopep-2 is a B3PP derived from Kunitz domains of human proteins (Zhou et al., 2021). Studies have reported the high transcytosis ability of angiopep-2, reaching levels of 3-fold higher of transcytosis than aprotinin and 50-fold higher than that of transferrin and lactoferrin when tested *in vitro* using BBB bovine capillary assay (Demeule et al., 2008). There are some applications of this peptide as BBB shuttle with conjugates, such as neurotensin and coumaric acid (Demeule et al., 2014; Suksrichavalit et al., 2014).

SynB is a cationic peptide derived from the naturally occurring antimicrobial peptide protegrin (Lalatsa et al., 2014). SynB1 and SynB3 peptides have been shown to have the ability to transport some drugs through the BBB by adsorptive-mediated endocytosis pathway (Drin et al., 2003). These peptides were conjugated with doxorubicin and showed a significant increase of drugs uptake into the brain, about 30-fold more than the molecule used solely (Rousselle et al., 2001). Similarly, benzylpenicillin and M6G also were tested as conjugated using SynB1 and they showed an increase of concentration in the brain (Liu et al., 2014). SynB1 was also conjugated with dalargin and presented an improvement in the delivery of this drug across the BBB and the analgesic activity of dalargin (Rousselle et al., 2003).

A summary of B3PPs applied in the development of new bioactive molecules against neurodegenerative disorders is shown in **Table 2**.

**Table 2:** List of names, primary structure, biological activities, and references for the cited peptides with therapeutic effect against neurodegenerative diseases, as well as BBB shuttle property.

Peptide names	Amino acid sequences	Biological activities	References
Ziconotide	CKGKGAKCSRLMYDCCTGSCRSGKC	Blocker of Ca^2+^ channel, preventing neuronal damage	(Zieminska et al., 2006)
Exenatide	HGEGTFTSDLSKQMEEEAVRLFIEWLKNGGPSSGAPPPS	Inhibitor of GLP-1R	(Hunter and Hölscher, 2012; Athauda and Foltynie, 2016; Athauda et al., 2017)
PhTx3-3	GKCADAWESCDNYPCCVVNGYSRTCMCSANRCNCDDTKTLREHFG	Blocker of Ca^2+^ channel, exocytosis, and glutamate release	(Pinheiro et al., 2009; Yang et al., 2019)
PhTx3-4	SCINVGDFCDGKKDCCQCDRDNAFCSCSVIFGYKTNCRCE	Blocker of Ca^2+^ channel, exocytosis, and glutamate release	(Pinheiro et al., 2009; Yang et al., 2019)
P110	YGRKKRRQRRRGGDLLPRGS	Inhibitor of DRP1	(Filichia et al., 2016)
Ba-V*	**-**	Inhibitor of mitochondrial permeability transition, preventing PD and AD	(Martins et al., 2010)
Transportan 10	AGYLLGKINLKALAALAKKIL	BBB shuttle of small molecules (e.g. dopamine and vancomycin)	(Rusiecka et al., 2019; Ruczyński et al., 2019)
Angiopep-2	TFFYGGSRGKRNNFKTEEY	BBB shuttle of small molecules (e.g. neurotensin and coumaric acid)	(Demeule et al., 2008; Suksrichavalit et al., 2014)
SynB1	RGGRLSYSRRRFSTSTGR	BBB shuttle of small molecules (e.g. doxorubicin, Benzylpenicillin, and M6G)	(Rousselle et al., 2003; Liu et al., 2014)
SynB3	RRLSYSRRRF	BBB shuttle of small molecules (e.g. doxorubicin,	(Drin et al., 2003)

*Ba-V is not only one peptide, but a family of peptides.

### Applications as Antimicrobial Agents

Antimicrobial peptides (AMPs) are a class of molecules that composes the innate defense of several species against pathogen infection and these structures have been considered as a useful strategy to combat antimicrobial resistance (Annunziato and Costantino, 2020). Multiple antibiotic resistance mechanisms have been described for bacterial cells and the resurgence of new mechanisms has been driven by the evolutionary processes that include gene mutations and natural selection (Browne et al., 2020).

AMPs have gained attention as a new class of antimicrobial compounds with the clinical potential to produce new antibiotics to combat resistant microorganisms. These peptides have been identified in a variety of species, where they act against pathogenic microorganisms (Diamond et al., 2009; Zhang and Gallo, 2016).

The AMPs have a high diversity of molecular mechanisms of action, where the most prevalent is the direct activity against the cell membrane (Pushpanathan et al., 2013). The ability to interact with these membranes is due to the amphipathic nature of these peptides, which are often positively charged, facilitating the electrostatic interaction between these cationic peptides with anionic cell membranes. These interactions induce the disruption of the cell membrane and insertion of AMPs, leading the cell death (Zhang and Gallo, 2016). Other classes of AMPs act against nucleic acid production and cell wall synthesis (Annunziato and Costantino, 2020).

Over the years, some studies have demonstrated antimicrobial activities of some peptides. For example, granulysin (GNLY) is produced by human cytolytic T lymphocytes, which have a broad spectrum of inhibitory activity against several Gram-positive and Gram-negative bacteria (Krensky, 2000). Mersacidin is another AMP that contains beta-methyllanthionine, which demonstrated inhibitory properties against the growth of several Gram-positive bacteria, including methicillin-resistant *Staphylococcus aureus* (MRSA). Its action against the MRSA is related to the binding to the cell wall precursor lipid II and blockage of cell wall biosynthesis (Chartterjee et al., 1992; Herzner et al., 2011).

The antimicrobial activity also has been reported in peptides extracted from snake venom. Recently, it has been demonstrated that the peptide OH-CATH is efficient against cephalosporin-resistant *Escherichia coli* (Bai-Yu et al., 2013). DEFB114 is another AMP found in the epididymis and gingival cells, that showed antimicrobial activity against several pathogenic microorganisms, such as *Staphylococcus aureus*, *Escherichia coli*, and *Candida albicans* (Yu et al., 2013).

Buforin II is under clinical trials and it has been explored due to its activity against different microorganisms. This peptide act through the accumulation into the cytoplasmatic medium and high-biding interactions with the nucleic acids, which leads to cell death. This peptide has shown a broad spectrum of antimicrobial activity against Gram-positive and Gram-negative bacteria, as well as fungi, such as *Candida albicans, Saccharomyces cerevisiae*, and *Cryptococcus neoformans* (Park et al., 1998; Cirioni et al., 2009). Recently, some peptides, such as omiganan (MX-226), novexatin (NP213), hLF(1-11), demegel (D2A21), and ETD151 also have demonstrated antifungal activities against drug-resistant strains of *Candida* sp. (Browne et al., 2020; Fernández de Ullivarri et al., 2020).

A summary of the cited peptides and other peptide-based antibiotics investigated regarding their antimicrobial activities is shown in **Table 3**.

**Table 3:** List of names, primary structure, biological activities, and references for the cited peptides with antimicrobial activity.

Peptide names	Amino acid sequences	Biological activities	References
GNLY	GRDYRTCLTIVQKLKKMVDKPTQRSVSNAATRVCRTGRSRWRDVCRNFMRRYQSRVIQG … LVAGETAQQICEDLR	Antibacterial	(Krensky, 2000)
Mersacidin	MSQEAIIRSWKDPFSRENSTQNPAGNPFSELKEAQMDKLVGAGDMEAACTFTLPGGGGVCTLTSECIC	Antibacterial activity	(Chartterjee et al., 1992; Herzner et al., 2011)
OH-CATH	MEGFFWKTLLVVGALAIGGTSSLPHKPLTYEEAVDLAVSIYNSKSGEDSLYRLLEAVPPPE … WDPLSESNQELNFTIKETVCLVAEERSLEECDFQEDGAIMGCTGYYFFGESPPVLVLTCK … PVGEEEEQKQEEGNEEEKEVEKEEKEEDEKDQPRRVKRFKKFFKKLKNSVKKRAKKFFK…KPRVIGVSIPF	Antibacterial activity	(Bai-Yu et al., 2013)
DEFB114	MRIFYYLHFLCYVTFILPATCTLVNADRCTKRYGRCKRDCLESEKQIDICSLPRKICCTEKLY…EEDDMF	Broad-spectrum antibacterial, antifungal activities	(Yu et al., 2013)
Buforin II	TRSSRAGLQWPVGRVHRLLRK	Broad spectrum antibacterial and antifungal activities	(Park et al., 1998; Fernández de Ullivarri et al., 2020)
Omiganan	H-*ξ*lle-LRWPWWPWRRK-NH_2_	Broad-spectrum antifungal, antibacterial	NCT02576847**
Novexatin	cyclo[RRRRRRR]	Antifungal activity	NCT02933879**
hLFroad-spectrum(1-11)	GRRRRSVQWCA	Broad-spectrum antibacterial and antifungal activities	NCT00509847**
Demegel	FAKKFAKKFKKFAKKFAKFAFAF	Antibacterial, antifungal	(Chalekson et al., 2002)
ETD151	DKLIGSCVWGAVNYTSNCRAECKRRGYKGGHCGSFANVNCWCET	Antifungal activity	(Browne et al., 2020)
Bacitracin*	Unk-L-dEIK(1)-dOrn-I-dFH-dDN-(1)	Antibacterial activity	(Browne et al., 2020)
Colistin*	Unk-Dab-T-Dab-Dab(1)-Dab-dLL-Dab-Dab-T(1)	Antibacterial activity	(Browne et al., 2020)
Polymyxin B*	Unk-Dab-T-Dab-Dab(1)-dDab-dFL-Dab-Dab-T(1)	Antibacterial activity	(Browne et al., 2020)

*Bacitrin, Colistin, and Polymyxin B are commercially available peptide-based antibiotics.

**The alphanumeric codes correspond to identifiers of the ClinicalTrials.gov of the US National Library of Medicine.

### Applications as Delivery Systems of Nucleic Acids and Drugs

CPPs structures have been investigated as vectors for nucleic acid molecules (Wolfe et al., 2018). Oligonucleotides conjugated with CPPs system carriers include short single- or double-stranded RNA and DNA molecules or its analog sequences, such as mRNAs, pre-mRNAs, and micro-RNAs which can target specific genes and modify the gene expression (Klabenkova et al., 2021).

Recently, there are several classes of therapeutic oligonucleotide mediated by peptides, such as antisense oligonucleotides (ASOs), small interfering RNAs (siRNAs), deoxyribozymes (DNAzymes), CRISPR/Cas9, antagomirs, and ribozymes (Taylor and Zahid, 2020; Klabenkova et al., 2021).

The siRNAs are double-stranded synthetic or natural oligoribonucleotides with a length of, 20–25 nt per strand, which can be used in a process of specific gene silencing named RNA interference (Taylor and Zahid, 2020). Although siRNAs have been used as a promising therapeutic agent with some approved drugs, such as givosiran (Givlaari (Scott, 2020) and lumasiran (Oxlumo^®^) (Scott and Keam, 2021), some hurdles regarding the internal metabolism of cells impacts the delivery of these molecules into the cytoplasm.

The CPPs have been also used as a mechanism of siRNA delivery to some tissues (Ruan et al., 2018) and organs (Li et al., 2014). However, cancer therapy became the main aim of studies that analyze the siRNA carried by peptides. Recently, amphipathic helical 12-mer peptides containing α,α-disubstituted α-amino acids (dAAs) were developed to deliver siRNA into living human hepatoma cells, and results showed that the conjugated peptide significantly increased of RNA interference (RNAi) into the cell (Furukawa et al., 2020). Recently, Lo et al. (2018) evaluated how tandem peptides combining CPP and iRGD can encapsulate siRNA to form a complex able to cross the desmoplastic stromal barrier, delivering this nucleic acid to pancreatic ductal adenocarcinoma cells (Lo et al., 2018). Another study explored the mechanism of the epidermal growth factor receptor (EGFR)-targeting peptide to deliver siRNA into EGFR-overexpressing oral cancer cells, aiming the silencing of the target oncogene (Alexander-Bryant et al., 2017). Similarly, Yang et al. (2016a) investigated how c-myc gene expression of breast cancer cells (MCF-7 cells) could be suppressed by a siRNA carrier system, which combines three components to perform the cell delivery: acid-sensitive polymer micelles, folic acid, and bio-reducible disulfide bond linked siRNA-CPPs conjugate (Yang et al., 2016a).

CPPs have also been investigated as system carriers of nanoparticles aiming for applications related to gene therapy. For example, nanoparticles composed of low-molecular-weight polyethylenimine and β-cyclodextrin were linked with folic acid and octa-arginine (R8) to deliver plasmid DNA to folate-receptor positive tumor cells, resulting in a high transfection level of this conjugate into cells (Jiang et al., 2011). Huang et al. (2013) investigated the use of tumor activatable CPP (termed dtACPP) to label nanoparticles and deliver siRNA targeting vascular endothelial growth factors. The experiments showed effective shutdown of blood vessels and cell apoptosis within the tumor (Huang et al., 2013).

Polymer nanoparticles modified with photo- and pH-responsive polypeptides (PPP) were developed to respond to near-infrared (NIR) light illumination at the tumor site and a lowered tumor extracellular pH (pHe). The results showed that the complex PPP-nanoparticles selectively accumulated at the tumor site and internalized into the tumor cells mediated by NIR light and the lowered pHe at the tumor region, demonstrating the potential of this complex as a carrier system in tumor gene therapy (Yang et al., 2016b). Mesken et al. (2017) developed a non-viral gene delivery system using plasmid-loaded human albumin nanoparticles, which were modified using CPPs, such as Tat, EB1, and nona-arginine (R9), then tested regarding their penetration into HEK 293T cells. The results showed a significant increase in the transfection level of these proposed vectors when compared with free DNA or polyplexes (Mesken et al., 2017).

## Computational Challenges and Future Developments

Different computational techniques have been developed to shed light on peptides’ pharmacokinetic properties, supporting studies that aim to investigate the molecular mechanism of translocation through the biomembranes and elucidate the physicochemical and structural properties associated with their molecular functions. The computational prediction offers an efficient and low-cost strategy to classify large compound libraries, but their analyses can show limitations regarding the applications and purposes of the study.

The MD simulations are examples of high computing enhanced sampling techniques that have been applied to demonstrate the conformational changes of peptides and their main biophysical interactions with biomembrane components, aiming to understand their mechanism of penetration into these membranes. However, this computational technique has been inappropriate to assess large compound libraries due to its high demand for time and computational processing. Thus, it is warranted new systematic studies to develop force fields with improved computational efficiency and accuracy that could better describe the molecular mechanism of penetration in these biomembranes, accessing in an appropriate time scale the peptide crossing. The development of membranes with alternative lipidic compositions has been also pointed out as relevant to reach a more realistic simulation, as well as to mimic biomembranes from different species (Marrink et al., 2019). More precise predictions of membrane crossing could be achieved by the use of multiple sampling techniques, with high-temperature conditions to speed up the kinetics involved with peptide folding on the membrane surface, thus assessing the penetration into the bilayer (Sugita et al., 2021).

With regards to machine learning algorithms, cheminformatic filters, and QSPR models, these techniques are useful and widely applied computational tools to screen large compound libraries and to understand the key structure- and sequence-based characteristics associated with the pharmacokinetics of these peptides. However, their predictive efficiency are also prone to biases and could lead to false identification of correct class of bioactive molecules.

The ML-based algorithms have been pointed out as a more efficient computational approach to predict hit compounds from large compound libraries than the traditional QSPR models (Tsou et al., 2020). However, further developments of ML-based algorithms could be related to the prediction of molecular mechanisms of penetration in the biomembranes, which currently can be accessed only by high computing analyses using MD simulations.

The limited information of peptides permeability in the online databases also impact negatively in the development of precise supervised ML-based algorithms that requires a comprehensive dataset composed of negative and positive ones to be trained. Furthermore, unfeasible ML-based models could be obtained using a training dataset composed of active molecules that are easily differentiated from inactive ones by coarse properties, such as HBA, HBD, logP, and MW (Ripphausen et al., 2011). Similarly, it has been demonstrated that ML-based models that use an optimized combination of structure- and sequence-based descriptors of peptides could show an improved performance than those that use only structure- or sequence-based descriptors separately (de Oliveira et al., 2021).

Highly correlated training and testing datasets of molecules, could also limit the applicability of some ML-models to classify correctly the active molecules, thus reaching to a false high training accuracy (Wallach and Heifets, 2018). Therefore, low training errors are insufficient to justify the choice of the most appropriate ML-based framework since the satisfactory predictive performance could be due to redundancy between the training and testing datasets rather than accuracy (Wallach and Heifets, 2018).

Currently, there are also limitation related to the prediction or assessment of the different conformational states acquired by the peptides. Chameleonic peptides can change their conformation to better cross the biological membranes (Matsson and Kihlberg, 2017). In addition, these peptides can also adopt multiple bioactive conformations in solution that are not satisfactory predicted only by the high throughput computational screening methods, such as QSPR models and ML algorithms (Miao et al., 2021). Currently, the chameleonic properties of peptides are only accessed using high computing sampling methods (Miao et al., 2021). Thus, due to the limited ability to predict complete structural ensembles of some peptides, which include, for example, the majority of macrocyclic peptides, we are also impaired to develop better strategies for the rational peptide design (Miao et al., 2021).

The development of new QSPR models and ML algorithms to predict the molecular mechanism of penetration of peptides in the biological membranes face some limitations due to the missing data of these molecular mechanisms in some databases (Gautam et al., 2012; Agrawal et al., 2016). Thus, the availability of experimental results in online databases related to the mechanism of penetration into biomembranes could provide more informative data to develop new robust computational tools applied in the prediction of pharmacokinetic properties of peptides, facilitating the discovery and development of new therapeutic agents based on these molecules.

## Final Considerations

Herein, we glimpsed the computational methods and some potential applications of CPPs and B3PPs, two classes of biomembrane-penetrating peptides which have been widely used for the development of new therapeutic molecules with neuroprotective, anticancer, and antimicrobial activities, as well as system carriers of molecules, such as nucleic acids and drugs. The myriad of their biotechnological and pharmaceutical applications has significantly increased over the years, as long as, new structural and conformational properties of these peptides are elucidated. The computational approaches applied to investigate their permeability into biomembranes are considered useful and cost-effective strategies that aim for scientific and technological purposes.

Concerning the ML-based algorithms, QSPR models, and cheminformatic filters, the development of more precise predictive models using these methods is highly dependent on the available data of these peptides in the online databases, thus increases of the experimental information regarding the physicochemical, structural properties, as well as their mechanism of penetration or existence of chameleonic properties will be useful for further developments.

Regarding the MD simulations, these methods offer a mechanist explanation of peptide crossing through the biomembranes which also could assist the development of molecules with improved pharmacokinetic properties or the development of more potent molecules delivery systems. However, continued attention has been given to the development of protocols that allies low time-consuming computational efforts and more precise predictive results. The development of these protocols has heavily relied on the existence of more realistic biophysical models of membranes that attend the particularities of their composition. Novel molecular forcefields and sampling methods conjugated with high-temperature simulations could also permit access to inappropriate time scale the molecular events related with peptides crossing through the biomembranes.

## Authors Contributions

Conceptualization was done by CS, KC and AL while investigation was headed by EO, KC, AL and PT. The original draft was prepared by KC, PT, CS and AL. Review and editing was done by CS, KC, AL and EO. All authors contributed to the article and approved the submitted version.

## Funding

AL, KC, and CS are grateful to Conselho Nacional de Desenvolvimento Científico e Tecnológico (CNPq, Brazil) for the financial support of the research; and EO is grateful to Coordenação de Aperfeiçoamento de Pessoal de Nível Superior (CAPES, Brazil) for the scholarship. All authors are grateful to Pró-reitoria de Pesquisa e Pós-graduação of Universidade Federal do Pará (PROPESP/UFPA) for the financial support.

## Conflict of Interest

The authors declare that the research was conducted in the absence of any commercial or financial relationships that could be construed as a potential conflict of interest.

## Publisher’s Note

All claims expressed in this article are solely those of the authors and do not necessarily represent those of their affiliated organizations, or those of the publisher, the editors and the reviewers. Any product that may be evaluated in this article, or claim that may be made by its manufacturer, is not guaranteed or endorsed by the publisher.
